# The Role of Prognostic Scores in Assessing the Prognosis of Patients Admitted in the Cardiac Intensive Care Unit: Emphasis on Heart Failure Patients

**DOI:** 10.3390/jcm13102982

**Published:** 2024-05-19

**Authors:** Aidonis Rammos, Aris Bechlioulis, Stefania Chatzipanteliadou, Spyros Athanasios Sioros, Christos D. Floros, Ilektra Stamou, Lampros Lakkas, Petros Kalogeras, Vasileios Bouratzis, Christos S. Katsouras, Lampros K. Michalis, Katerina K. Naka

**Affiliations:** Second Department of Cardiology, Faculty of Medicine, School of Health Sciences, University of Ioannina and University Hospital of Ioannina, 45110 Ioannina, Greece; a.rammos@uoi.gr (A.R.); md02798@yahoo.gr (A.B.); chatzipanteliadou.s@gmail.com (S.C.); spsior@yahoo.com (S.A.S.); christos.floros@yahoo.com (C.D.F.); ilektst@gmail.com (I.S.); ftpcavalier52@gmail.com (L.L.); pkalog90@yahoo.com (P.K.); v.bouratzis@gmail.com (V.B.); chkatsou@uoi.gr (C.S.K.); lamprosmihalis@uoi.gr (L.K.M.)

**Keywords:** critical care, cardiac intensive care unit, prognostic risk scores, in-hospital mortality, acute cardiac disease, advanced cardiac therapies

## Abstract

**Background/Objectives**: Patient care in Cardiac Intensive Care Units (CICU) has evolved but data on patient characteristics and outcomes are sparse. This retrospective observational study aimed to define clinical characteristics and risk factors of CICU patients, their in-hospital and 30-day mortality, and compare it with established risk scores. **Methods**: Consecutive patients (*n* = 294, mean age 70 years, 74% males) hospitalized within 15 months were studied; APACHE II, EHMRG, GWTG-HF, and GRACE II were calculated on admission. **Results**: Most patients were admitted for ACS (48.3%) and acute decompensated heart failure (ADHF) (31.3%). Median duration of hospitalization was 2 days (IQR = 1, 4). In-hospital infection occurred in 20%, 18% needed mechanical ventilation, 10% renal replacement therapy and 4% percutaneous ventricular assist devices (33%, 29%, 20% and 4%, respectively, for ADHF). In-hospital and 30-day mortality was 18% and 11% for all patients (29% and 23%, respectively, for ADHF). Established scores (especially APACHE II) had a good diagnostic accuracy (area under the curve-AUC). In univariate and multivariate analyses in-hospital intubation and infection, history of coronary artery disease, hypotension, uremia and hypoxemia on admission were the most important risk factors. Based on these, a proposed new score showed a diagnostic accuracy of 0.954 (AUC) for in-hospital mortality, outperforming previous scores. **Conclusions**: Patients are admitted mainly with ACS or ADHF, the latter with worse prognosis. Several patients need advanced support; intubation and infections adversely affect prognosis. Established scores predict mortality satisfactorily, but larger studies are needed to develop CICU-directed scores to identify risk factors, improve prediction, guide treatment and staff training.

## 1. Introduction

Critical care involves the diagnosis and management of life-threatening medical conditions that require close and continuous monitoring and is inherent to cardiovascular medicine [1]. The aging population, the complex and multiple comorbidities, the significant increase in the prevalence of heart failure (HF) and the evolution of advanced cardiovascular therapies (complex percutaneous coronary intervention, transcatheter aortic valve implantation, mitral and tricuspid valve repair, device implantation, temporary mechanical circulatory support, long-term ventricular assist device, in addition to many novel medications) which has led cardiovascular conditions previously regarded as terminal, to improved survival [2], has increased the need for cardiac critical care. There is expanding evidence that outcomes are improved when critical patients are treated in dedicated Cardiac Intensive Care Units (CICU) [1]. However, patients admitted to CICU show a great variability regarding the reason for admission and patient profile and thus patients’ prognosis and clinical outcomes also vary significantly.

In order to guide clinicians in providing a more effective care and aid prediction of adverse outcomes, several risk models, like the Acute Physiology and Chronic Health Evaluation (APACHE) scoring system have been developed mainly in the general ICU population [3]. In contrast, there are few risk scores available for CICU patients, and most of them refer to disease-specific populations such as the Get with The Guidelines—Heart Failure (GWTG-HF) and the Emergency Heart Failure Mortality Risk Grade (EHMRG) scores for patients admitted with acutely decompensated heart failure (ADHF) or the GRACE II score for patients with an acute coronary syndrome (ACS). Some of the ICU risk scores (e.g., the APACHE score) have also been used to predict outcomes in CICU patients [3], although problems exist regarding their validity and applicability, as they have been developed and validated in non-CICU populations [4]. Therefore, there seems to be an emerging need for validated tools that can be used in CICU patients in every day clinical practice, to provide important information regarding the prognosis and management of these patients.

In the current retrospective observational study, we aimed to define the clinical characteristics of patients admitted in a CICU of a tertiary University Hospital and identify on-admission factors that could assist the prediction of in-hospital and short-term mortality as well as to assess the applicability of established risk scores in this population.

## 2. Materials and Methods

The present study is a single-center, retrospective, observational study that enrolled 294 consecutive patients admitted to the CICU within 15 months (from January 2022 to March 2023) of a Greek tertiary University Hospital with a 10-bed CICU. The CICU is staffed with a General Cardiology Fellow under the supervision of a general and an interventional cardiologist. Patients were either admitted to the CICU from the Emergency Department or transferred from other secondary regional hospitals. Patients with complicated, high-risk ACS (i.e., patients perceived to be at higher risk compared to those usually admitted to an ordinary Coronary Care Unit) were admitted to the CICU, as well as patients who were at need for intensive cardiac care because of an acute cardiac condition. The various acute cardiac conditions of admitted patients were classified into the following groups: acute coronary syndromes (ACS), acute de novo or acutely decompensated heart failure (ADHF), pulmonary embolism (PE) with hemodynamic instability, arrhythmias (sustained ventricular tachycardia or high degree atrioventricular block), and other acute cardiovascular pathologies (i.e., acute myocarditis, cardiac tamponade, acute aortic syndromes etc.). Patients who stayed in CICU for less than 24 h (i.e., for post-procedural monitoring, stable CAD, pericardial diseases other than tamponade etc.) or patients who may have been admitted to the CICU for various non-medical reasons (local practice, social reasons, provider preferences etc.) have been excluded from this study, in order to include only patients who are at truly high cardiovascular risk and at need of cardiac intensive care.

In all enrolled patients a wide dataset of variables was recorded on admission and four established risk scores were calculated: APACHE II, GWTG-HF, EHMRG and GRACE II. Patients were followed during their hospitalization in the CICU and the cardiology ward until discharge. The occurrence of various complications during CICU stay (such as sepsis/infections, cardiac arrest, acute respiratory failure, acute kidney failure) that led to major interventions (i.e., mechanical ventilation, mechanical circulatory support, renal replacement therapy etc.) were also recorded. Mortality during hospitalization or at 30 days, 6 months and 12 months were the outcomes of interest. A telephone interview was planned at each of these time-points. In patients who were discharged from the hospital, other outcomes such as non-fatal myocardial infarctions or strokes, ADHF re-hospitalization and other cardiovascular hospitalizations, were also recorded. At follow-up, 54 patients died in hospital, 53 patients died after discharge and during the 12 month period, while 124 patients presented at least one of the above described major adverse cardiovascular events during the same period; 7 patients were lost to follow-up.

The study protocol was approved by the local Ethics Committee and informed consent was obtained from all enrolled patients. The study conformed to the principles outlined in the Declaration of Helsinki.

### Statistical Analysis

Continuous variables are presented as mean ± standard deviation or median (interquartile range—IQR) and dichotomous variables are presented as number (percentage). Univariate associations of various studied parameters with in-hospital and 30-day mortality were assessed using logistic regression analysis. Multivariate analysis was performed using backward conditional logistic regression analysis and included parameters with a univariate association at *p* < 0.1 level of statistical significance. Predicted probabilities from multivariate regression models were saved. Receiver operating characteristic curve (ROC) analysis was used to assess the predictive accuracy of the well-known risk scores as well as the multivariate regression models generated from our population. The risk scores for the prediction of outcomes were created on the basis of the results of the Multivariate Logistic Regression analysis. The points for each variable included in the scores were associated with the corresponding value of Odds Ratio (OR) in the Regression model; the parameters with the lower OR values in the regression model scored 1 point each while parameters with higher values scored 2–3 points depending on the magnitude of their OR. *p* values were always two-sided and a value of *p* < 0.05 was considered significant. The SPSS statistical software package (IBM Corp Released 2015. IBM SPSS Statistics for Windows, Version 23.0, IBM Corp, Armonk, NY, USA) was used.

## 3. Results

### 3.1. Analysis of In-Hospital Mortality in All Patients

The descriptive data of the recorded parameters on admission of the enrolled patients are shown in Table 1. The main reasons for admission to CICU were an ACS (48%) and ADHF (31%). Other reasons for CICU hospitalization were major PE (2%), arrhythmias (12%), and other causes in 7% of patients (i.e., cardiac tamponade, myocarditis, acute aortic syndromes etc.). The median duration of stay in CICU was 2 days. Fifty-four patients (18%) died prior to hospital discharge.

Univariate analysis associations of various studied parameters with in-hospital mortality in the entire population are shown in Table 2. In multivariate analysis, need for mechanical ventilation (OR 43.52, *p* < 0.001), in-hospital infection (OR 4.42, *p* < 0.001), previous history of CAD (OR 4.20, *p* = 0.008), low systolic blood pressure on admission (i.e., <100 mmHg) (OR 4.78, *p* = 0.002), low SatO_2_ < 90% on admission (OR 4.67, *p* = 0.021) and increased urea (>100 mg/dL) on admission (OR 2.97, *p* = 0.038) were independently associated with in-hospital mortality (Table 3).

The used risk scores predicted in-hospital mortality with a relatively good accuracy as assessed by the area under the curve (AUC): APACHE II 0.861, EHMRG 0.83, GWTG-HF 0.79 and GRACE II 0.819 (*p* < 0.001 for all). The regression model generated from the current population using the six variables included in the multivariate analysis (Table 3) predicted in-hospital mortality with an AUC 0.954, *p* < 0.001. A clinical score derived from this model was constructed based on the weight of OR values for each of the six parameters: three points for mechanical ventilation and one point for every other variable. A cut-off of total score > 2 could predict with sensitivity 91% and specificity 90% in-hospital mortality; patients with total score ≤ 2 and >2 showed 2.3% and 67.1% in-hospital mortality, respectively.

### 3.2. Analysis of In-Hospital Mortality in Patients Admitted for ADHF

The descriptive data and the recorded parameters on admission for the subgroup of patients admitted for ADHF are presented in Table 1. Univariate analysis associations of various studied parameters with in-hospital mortality in the subgroup of patients admitted for ADHF are shown in Table 2. In multivariate analysis, need for mechanical ventilation (OR 68.39, *p* < 0.001), in-hospital infection (OR 9.29, *p* < 0.015), history of CAD (OR 10.01, *p* = 0.016), low mean blood pressure < 60 mmHg on admission (OR 5.81, *p* = 0.033), low SatO_2_ < 90% on admission (OR 11.73, *p* = 0.042) and increased urea > 100 mg/dL on admission (OR 10.55, *p* = 0.015) were independently associated with in-hospital mortality (Table 3).

In the subgroup of patients admitted for ADHF, the used risk scores predicted in-hospital mortality with a relatively good accuracy as assessed by the AUC: APACHE II 0.805, EHMRG 0.775, GWTG-HF 0.767 and GRACE II 0.719 (*p* < 0.001 for all). The regression model generated from the current population using the six variables included in the multivariate analysis (Table 3) predicted in-hospital mortality with an AUC 0.964, *p* < 0.001.

### 3.3. Analysis for 30-Day Mortality in Patients Discharged from the Hospital

The descriptive data and the recorded parameters on admission in the patients discharged alive from hospital (*n* = 233) are shown in Table 1. Twenty-five patients (11%) died during the first month after discharge; of those, fifteen patients had been initially admitted for ADHF. Univariate analysis associations of various studied parameters with 30-day mortality in the entire group of patients discharged from the hospital as well as the subgroup of patients discharged with the original diagnosis of ADHF are shown in Table 2. In multivariate analysis, in-hospital infection (OR 3.85, *p* = 0.008), ADHF as the reason of admission (OR 3.12, *p* = 0.015) and a ratio PO_2_/FiO_2_ < 300 (OR 3.55, *p* = 0.021) were independently associated with 30-day mortality in all patients discharged from the hospital (Table 3). In the subgroup of patients with diagnosis of ADHF on admission, independent predictors of 30-day mortality were in-hospital infection (OR 7.04, *p* = 0.008) and low mean arterial blood pressure < 60 mmHg (OR 6.67, *p* = 0.014) (Table 3).

The used risk scores predicted 30-day mortality in patients discharged from the hospital alive, with a moderate accuracy; AUC was for: APACHE II 0.795, EHMRG 0.674, GWTG-HF 0.712 and GRACE II 0.684 (*p* < 0.01 for all). The regression model generated from the current population using the three variables included in the multivariate analysis (Table 3) predicted 30-day mortality with an AUC 0.786, *p* < 0.001. In the subgroup pf patients discharged alive with the diagnosis of ADHF, only the APACHE II score predicted 30-day mortality and with a low accuracy AUC 0.682, *p* < 0.04. The regression model generated from the current population using the two variables included in the multivariate analysis (Table 3) predicted 30-day mortality with an AUC 0.786, *p* = 0.004.

## 4. Discussion

In the current retrospective observational study, a substantial number of patients admitted to the CICU died in the following 12 months (i.e., 107/294 patients, 36.4%) with approximately half of them dying during the index hospitalization. This finding indicates that, irrespective of the patient’s profile, the need for CICU admission per se is a marker of severe underlying conditions with high mortality. The majority of hospitalized patients were admitted with an ACS or ADHF and the median length of stay in the CICU was 2 days, similar to previous studies [5,6,7]. In-hospital mortality rates were found to be lower compared to previously published data [6,8].

However, data on CICU patients is limited and there is great variability regarding the population characteristics, the setting, as well as the patient’s care that has improved in modern times. Historically, there is a gradual decline in the percentage of admissions for ACS and an increase in ADHF admissions, while patients admitted to the CICU are older with multiple comorbidities and various non-cardiac complications such as infections/sepsis, acute kidney injury and respiratory failure; these highlight the epidemiological changes in cardiovascular disease over the last decades, advocating the transition from a coronary care unit to a CICU covering many different acute cardiac pathologies [9]. According to recent data, 98% of hospitals in Europe have a dedicated CICU; 70% have a first level unit treating medical conditions demanding low levels of intensive care, 76% have a second level unit, able to provide moderate levels of care, while 51% have a third level unit treating patients with acute cardiac conditions severe enough or highly probable to require mechanical circulatory, renal or pulmonary support [5]. CICU staffing appears to have great differences among countries; the most striking of those appears to be the availability of nursing and medical personnel per patient [10]. CICUs that are staffed by more experienced nursing personnel and cardiac intensivists appear to have lower rates of CICU or hospital mortality and length of stay [1,11,12,13], even when the severity of illness is higher [14]. 

The risk scores currently used to predict mortality in patients admitted to ICU showed a moderate to high diagnostic accuracy, as assessed by the AUC in ROC curve analysis for in-hospital and 30-day mortality. Interestingly, the APACHE II score, although built in a different setting and population in the general ICU [3], showed the highest discriminative power. On the other hand, the scores built from “cardiological” populations, either HF or ACS, were shown to have an inferior performance compared to APACHE. 

The risk score for in-hospital mortality derived from the multivariate regression model applied in the current study population seemed to outperform all other risk scores; however, overfitting errors have to be considered and validation in other CICU populations and larger samples are needed. Interestingly, the score was based on features related to the patient’s status on admission to the CICU (low blood pressure, impaired oxygenation, increased urea) and short-term complications (need for invasive ventilation and infection during hospitalization), while the patient’s medical history did not appear to play a very important role. The general principles on which the score derived from the current study was based, are similar to those used for the APACHE II risk score that has been previously shown to have a high predictive accuracy for in-hospital mortality (AUC 0.90) among CICU patients (the overwhelming majority of whom were patients with ACS) [15]. The risk score for in-hospital mortality developed from the current study did not include the reason of admission to the CICU (i.e., ACS vs. ADHF or other). ADHF (vs other reasons for admission) has been previously shown to be an independent predictor of 30-day mortality in patients who were discharged alive from the hospital [16]. 

In CICU patients, major complications that are known to prolong the duration of stay and increase adverse outcomes are: respiratory failure (in up to 30% of admitted patients), acute kidney injury often requiring renal replacement therapy (in up to 30%), and sepsis [7,17]. In the current study, respiratory failure in need for invasive mechanical ventilation was the strongest factor related to in-hospital mortality. Importantly, 42 out of the 53 patients who were intubated died in hospital (79%); this mortality rate is significantly higher than the overall mortality rate of 30%, reported in a meta-analysis for ICU intubated patients [18]. This difference may be related to the variability in the population characteristics (age, heart failure patients, and multiple comorbidities) but also in the training of the CICU staff in the management of intubated patients. In our population, invasive mechanical ventilation, although performed to save patients’ lives, is associated with a very high mortality risk. Whether this is related to the underlying patients’ risk (that led them to the intubation) or to complications arising whilst patients are intubated, should be studied further to guide training of nursing and medical staff in the CICU. In addition, whether other measures used to prevent or delay intubation, such as non-invasive ventilation, could reduce mortality risk in selected patients, should be explored in future research. Although the prognostic effect of invasive mechanical ventilation is grave during hospitalization, there is no carry-on effect in patients who were discharged alive. However, moderate-severe respiratory failure on-admission was shown to be an independent predictor of 30-day mortality in discharged patients. 

In our study, in-hospital infection occurred in 58 patients (20%); this was associated with an increase in the risk of in-hospital mortality by 4 times, irrespective of other patient’s characteristics. In fact, patients who were discharged alive but had suffered an in-hospital infection, carried an adverse prognosis even at 30-days follow-up. Sepsis has been reported to affect 16–37% of CICU patients, with a high risk of mortality, up to 44%, depending on the population and setting [19,20,21]. Patients with an ACS complicated by sepsis experience 103% higher odds of death compared to non-septic AMI patients [22]. In fact, in our subgroup of ACS patients who were at very high risk, 47.1% died in hospital due to sepsis. Non-cardiac multi-organ failure has also been reported to be significantly more prevalent in sepsis cohorts [23]. Similar to respiratory failure requiring mechanical ventilation, in-hospital infection emerges as a serious complication that should be avoided in order to reduce mortality. All hospital departments, and especially CICU and ICU, should establish effective practices and prevention measures to reduce in-hospital infections. 

Regarding patients’ medical history, prior CAD was the only medical history-related parameter that emerged as an independent risk factor, indicating that CAD patients admitted to CICU for any acute cardiac condition, are of higher risk compared to cardiac patients without a previous diagnosis of CAD. Finally, three parameters that are readily available on admission, i.e., systolic blood pressure < 100 mmHg, oxygen saturation < 90% and blood urea > 100 mg/dL, may be used to flag patients that are at very high-risk for death, guiding clinical decisions. More research is needed to investigate whether various types of interventions that can be used to correct these abnormalities may have a differential impact on patient prognosis. Interestingly, in the ADHF sub-group, low mean blood pressure < 60 mmHg was included among the predictors of mortality, instead of the low systolic blood pressure, probably indicating the importance of adequate renal perfusion in this group of patients. 

It is important to note that the predictive ability of the score derived from the current study population, as well as all other established scores, was largely reduced for the short-term period of 30 days. More research is needed to investigate this vulnerable period of time following discharge, and aid physicians in providing effective post-discharge follow-up and management.

Multi-organ complications in CICU patients and the complexity superimposed primarily by in-hospital infections highlight the need of a multidisciplinary approach and the presence of well-educated and trained CICU staff who are familiar with advanced cardiac critical care medicine [13,24,25,26]. Evidence derived from the ICUs has showed that hospitals that have adopted an organizational culture supportive of integrated care delivery provide optimal care [12], while staffing by critical care physicians has been associated with decreased mortality and reduced lengths of hospital stay, medical complications and health care costs [27]. However, even in newly developed CICUs, only a minority of the staff has been reported to possess intensivist skills [28]. The recent Clinical Practice Guidelines on Heart Failure-Related Cardiogenic Shock from the International Society of Heart and Lung Transplantation [29] suggest that, in a CICU, it is more effective to focus on available necessary roles, i.e., a clinician able to provide critical care, a clinician able to place mechanical circulatory support devices and perform cardiothoracic surgical procedures, a heart failure practitioner, a nursing health professional, a device specialist, palliative care, and pharmacy, than on distinct disciplines.

For the purposes of the current study, only patients who were at high cardiovascular risk and at true need of cardiac intensive care were included, acknowledging thus, that systematic overuse of CICUs for cardiac patients who do not immediately require therapies that are offered only in CICU should be discouraged. It is widely known that early reperfusion strategy and improvements in medical treatment, have greatly reduced in-hospital cardiac arrest among patients with non-ST segment-elevation myocardial infarction. These patients can thus be treated in intermediate coronary care units after an uncomplicated percutaneous coronary intervention [30]. Regarding the heart failure patient population, data on CICU use is limited; among ADHF patients, the incidence of hospital complications that may require a CICU admission has been previously reported to be approximately 12% [31].

Finally, the interpretation of the results of the current study should take into account various organizational issues and social needs that may be country-specific or hospital-specific and are usually related to the management of patients with advanced chronic diseases or end-of-life cases. An important issue, especially for the heart failure patient population, is whether CICU or ICU should be used for palliative care or end-of-life management in critical care patients, in the lack of other facilities. In our registry, a small percentage of HF patients (*n* = 6, 2.0%) died in the CICU, while receiving comfort measures only during a long CICU stay (median hospitalization length was 17 days, ranging from 1 to 32 days), due to either lack of supporting environment or social service shortcomings. In a North American registry, 68% of patients who died in CICU had received comfort measures only [32]. Patients with advanced HF often spend long time periods in the CICU, and palliative care is fundamental to meet the patients’ and family physical and emotional needs and also to reduce invasive and costly interventions that are not expected to benefit the patient or improve the quality of their remaining time [33,34,35].

## 5. Limitations

This was a single-center study in a specific setting of a local tertiary academic center and thus the results may not be applicable or generalizable to other settings. Although the risk scores used had inherent limitations (disease-specific, population variability, ICU population), they displayed a relatively good predictive accuracy for in-hospital mortality. Only on-admission parameters were used to risk-stratify patients and not the progress of these parameters during the hospitalization or discharge values which may also provide useful information. For this reason, data analysis for 6-month and 12-month outcomes was not performed in the current study. Finally, echocardiographic assessment (early, on-admission or longitudinal) was not included in our analysis or in any other established risk scores. However, a CICU-specific score developed exclusively for cardiac patients should probably include echocardiographic parameters as this approach may be expected to improve risk stratification, guide clinical decisions and affect in-hospital outcomes. 

## 6. Conclusions

Patients admitted to the CICU carry an increased risk for in-hospital mortality independent of the reason for admission. Admission characteristics and in-hospital complications were the main determinants of in-hospital mortality. Non-specific risk scores, derived from the general ICU, calculated on admission, were shown to predict in-hospital mortality with moderate accuracy and may be useful in the current management of CICU patients. However, a new risk stratification tool specifically built for CICU patients is needed to identify early high-risk patients who are candidates for advanced therapies and assist clinicians in everyday clinical decisions. Multidisciplinary teams with critical care experience and training are urgently needed to improve CICU patients’ care and outcomes.

Based on the results of this retrospective study, larger prospective research studies should be performed to provide the cardiology and medical community with robust data on the needs of patients admitted to a CICU and indicate how medical care and cardiac intensive training should be organized in order to cover these needs in the future.

## Figures and Tables

**Table 1:** Demographic, clinical and other on-admission characteristics of all patients and the ADHF subgroup.

	In-Hospital Analysis		Discharged Patients	
	All Patients (*n* = 294)	ADHF Group (*n* = 92)	All Patients (*n* = 233)	ADHF Group (*n* = 65)
Male gender, *n* (%)	218 (74)	61 (66)	171 (73)	42 (65)
Age, years	70 ± 14	74 ± 15	69 ± 15	74 ± 16
COPD, *n* (%)	51 (17)	27 (29)	38 (16)	18 (28)
GFR-EPI mL/min/1.73 m^2^	59.03 ± 27.86	44.70 ± 25.29	63.49 ± 26.87	50.58 ± 25.87
GFR < 60 mL/min/1.73 m^2^	143 (49)	66 (72)	98 (42)	41 (63)
ESRD, *n* (%)	11 (4)	4 (4)	9 (4)	4 (6)
Diabetes mellitus, *n* (%)	103 (35)	35 (38)	74 (32)	21 (32)
CAD, *n* (%)	93 (32)	35 (38)	68 (29)	21 (32)
Cancer active, *n* (%)	26 (9)	9 (10)	22 (9)	7 (11)
*On Admission recorded parameters*				
Systolic BP, mmHg	119 ± 29	110 ± 30	124 ± 25	115 ± 27
Mean BP, mmHg	83 ± 21	76 ± 20	87 ± 18	80 ± 19
Heart rate, bpm	79 (70, 95)	88 (75, 108)	77 (68, 92)	82 (72, 99)
Respiratory rate, bpm	15 (15, 16)	16 (15, 18)	15 (15, 16)	16 (15, 18)
Killip Category 4	41 (14)	19 (20)	15 (6)	6 (9)
SatO_2_, %	96 (93, 97)	95 (90, 97)	96 (94, 97)	95 (91, 97)
PaO_2_, mmHg	81 (72, 91)	90 (69, 106)	81 (72, 90)	51 (70, 107)
PaCO_2_ (mmHg)	37.5 ± 9.1	38.4 ± 11.8	37.0 ± 8.3	38.4 ± 11.5
HCO_3_^−^ (mEq/L)	21.4 ± 4.1	20.9 ± 4.3	21.9 ± 3.8	21.5 ± 4.0
PO_2_/FiO_2_	294 (184, 380)	220 (143, 323)	329 (212, 386)	249 (177, 338)
Lactate (mmol/L)	1.1 (0.8, 2.1)	1.5 (1.1, 2.4)	1.0 (0.8, 1.7)	1.4 (0.9, 2.1)
pH	7.40 (7.34, 7.40)	7.37 (7.30, 7.40)	7.40 (7.36, 7.41)	7.37 (7.32, 7.40)
Na^+^ (mEq/L)	137 ± 5	136 ± 6	137 ± 4	137 ± 5
K^+^ (mEq/L)	4.38 ± 0.72	4.46 ± 0.87	4.34 ± 0.68	4.43 ± 0.91
Creatinine (mg/dL)	1.15 (0.93, 1.62)	1.55 (1.10, 2.50)	1.09 (0.90, 1.45)	1.30 (1.05, 1.91)
Urea (mg/dL)	54 (38, 84)	78 (56, 137)	47 (36, 74)	69 (47, 111)
HsTroponin I (ng/L)	360 (54, 8463)	174 (59, 635)	306 (39, 6053)	105 (42, 386)
Hematocrit (%)	38.8 ± 7.1	37.4 ± 7.4	38.9 ± 7.2	37.5 ± 7.6
WBC × 10^3^/μL	10.03 (7.86, 13.70)	9.71 (7.25, 13.70)	10.03 (7.76, 13.19)	10.15 (7.18, 13.79)
*Other features recorded during CICU stay*				
In-hospital Arrest, *n* (%)	30 (10)	8 (9)	10 (4)	2 (3)
IMV, *n* (%)	53 (18)	27 (29)	12 (5)	7 (11)
MCS, *n* (%)	13 (4)	4 (4)	5 (2)	1 (2)
CVVHDF, *n* (%)	28 (10)	18 (20)	10 (4)	6 (9)
In-hospital infection, *n* (%)	58 (20)	30 (33)	30 (13)	13 (20)
Blood cultures (+), *n* (%)	33 (11)	17 (19)	15 (6)	6 (9)
Days in CICU	2 (1, 4)	3 (2, 7)	77 (33)	26 (40)
Mortality, *n* (%)	54 (18)	27 (29)	25 (11)	15 (23)
Risk Scores calculated				
APACHE II	12.0 (6.8, 17.0)	16.0 (13.0, 21.8)	11.0 (6.0, 15.0)	15.0 (12.0, 17.0)
EHMRG	86.8 (50.4, 139.8)	128.4 (80.6, 172.4)	77.9 (44.2, 121.0)	96.5 (65,8, 151.7)
GWTG-HF	50 (41, 59)	58 (49, 72)	47 (40, 56)	56 (48, 63)
GRACE II	142 (110, 170)	163 (142, 183)	137 (105, 163)	159 (140, 175)

ADHF = acute decompensated heart failure, BP = blood pressure, bpm = beats per minute, CAD = coronary artery disease, CICU = cardiac intensive care unit, COPD = chronic obstructive pulmonary disease, CVVHDF = continuous venovenous hemodiafiltration, ESRD = end stage renal disease, GFR = glomerular filtration rate, IMV = invasive mechanical ventilation, MCS = mechanical circulatory support, WBC = white blood cells.

**Table 2:** Univariate associations of studied parameters with in-hospital and 30-day mortality in all patients and the ADHF subgroup.

	In-Hospital Analysis		Discharged Patients	
	All Patients (*n* = 294)	ADHF Group (*n* = 92)	All Patients (*n* = 233)	ADHF Group (*n* = 65)
Male gender	OR 1.27, *p* = 0.501	OR 1.30, *p* = 0.595	OR 0.75, *p* = 0.520	OR 2.67, *p* = 0.165
Age/5 years increase	OR 1.10, *p* = 0.098	OR 0.99, *p* = 0.883	OR 1.25, *p* = 0.01	OR 1.09, *p* = 0.434
Age ≥ 70 years-old	OR 1.54, *p* = 0.165	OR 0.77, *p* = 0.589	OR 2.47, *p* = 0.052	OR 1.07, *p* = 0.919
COPD	OR 1.69, *p* = 0.152	OR 1.31, *p* = 0.589	OR 2.22, *p* = 0.101	OR 0.94, *p* = 0.919
GFR-EPI/15 mL/min^2^	OR 0.59, *p* < 0.001	OR 0.55, *p* = 0.001	OR 0.82, *p* = 0.084	OR 1.19, *p* = 0.315
GFR < 60 mL/min/1.73 m^2^	OR 5.47, *p* < 0.001	OR 7.32, *p* = 0.011	OR 1.88, *p* = 0.140	OR 0.59, *p* = 0.375
Diabetes mellitus	OR 2.16, *p* = 0.012	OR 2.26, *p* = 0.082	OR 0.82, *p* = 0.670	OR 1.06, *p* = 0.923
CAD	OR 1.98, *p* = 0.027	OR 2.26, *p* = 0.082	OR 0.58, *p* = 0.290	OR 0.44, *p* = 0.253
Cancer, active	OR 0.79, *p* = 0.681	OR 0.66, *p* = 0.623	OR 2.01, *p* = 0.243	OR 1.39, *p* = 0.716
ADHF group	OR 2.69, *p* = 0.001	-	OR 4.74, *p* < 0.001	-
SBP/15 mmHg increase	OR 0.61, *p* < 0.001	OR 0.72, *p* = 0.012	OR 0.87, *p* = 0.265	OR 0.93, *p* = 0.647
SBP < 100 mmHg	OR 10.59, *p* < 0.001	OR 4.80, *p* = 0.001	OR 1.83, *p* = 0.266	OR 1.58, *p* = 0.473
MBP/10 mmHg increase	OR 0.61, *p* < 0.001	OR 0.69, *p* = 0.007	OR 0.79, *p* = 0.051	OR 0.74, *p* = 0.086
MBP < 60 mmHg	OR 11.23, *p* < 0.001	OR 5.29, *p* = 0.001	OR 4.37, *p* = 0.007	OR 6.00, *p* = 0.011
HR/10 bpm increase	OR 1.38, *p* < 0.001	OR 1.43, *p* = 0.002	OR 1.05, *p* = 0.643	OR 0.91, *p* = 0.508
HR > 100 bpm	OR 4.07, *p* < 0.001	OR 3.59, *p* = 0.008	OR 1.05, *p* = 0.936	OR 0.44, *p* = 0.317
RR/1 breath increase	OR 1.12, *p* = 0.003	OR 1.07, *p* = 0.193	OR 1.17, *p* = 0.003	OR 1.08, *p* = 0.265
RR > 15 brpm	OR 2.18, *p* = 0.013	OR 1.21, *p* = 0.697	OR 3.31, *p* = 0.01	OR 1.88, *p* = 0.375
SatO_2_/2% increase	OR 0.83, *p* = 0.002	OR 0.91, *p* = 0.233	OR 0.88, *p* = 0.125	OR 1.14, *p* = 0.328
SatO_2_ < 90%	OR 4.76, *p* < 0.001	OR 2.62, *p* = 0.082	OR 2.23, *p* = 0.241	OR 0.38, *p* = 0.375
PaO_2_/20 mmHg increase	OR 1.05, *p* = 0.573	OR 0.96, *p* = 0.703	OR 1.20, *p* = 0.104	OR 1.30, *p* = 0.049
PaCO_2_/1 mmHg increase	OR 1.03, *p* = 0.032	OR 0.99, *p* = 0.703	OR 1.00, *p* = 0.996	OR 1.01, *p* = 0.576
HCO_3_^−^/3 mmol/L increase	OR 0.61, *p* < 0.001	OR 0.70, *p* = 0.030	OR 0.99, *p* = 0.978	OR 1.58, *p* = 0.084
PO_2_/FiO_2_/50 increase	OR 0.62, p < 0.001	OR 0.74, *p* = 0.01	OR 0.72, *p* = 0.001	OR 1.04, *p* = 0.756
PO_2_/FiO_2_ < 300	OR 7.16, *p* < 0.001	OR 2.41, *p* = 0.116	OR 6.03, *p* = 0.001	OR 1.69, *p* = 0.424
Lactate (mmol/L)				
1.0–2.0 (mmol/L)	OR 2.61, *p* = 0.052	OR 1.56, *p* = 0.548	OR 3.13, *p* = 0.046	OR 1.96, *p* = 0.446
>2.0 (mmol/L)	OR 12.49, *p* < 0.001	OR 4.77, *p* = 0.028	OR 6.25, *p* = 0.002	OR 4.38, *p* = 0.097
pH				
7.35–7.40	OR 2.14, *p* = 0.08	OR 1.22, *p* = 0.761	OR 1.94, *p* = 0.205	OR 0.76, *p* = 0.714
<7.35	OR 5.76, *p* < 0.001	OR 2.20, *p* = 0.163	OR 2.32, *p* = 0.096	OR 0.67, *p* = 0.558
Na^+^/5 mmol/L increase	OR 0.72, *p* = 0.032	OR 0.61, *p* = 0.017	OR 0.69, *p* = 0.117	OR 0.85, *p* = 0.562
Na^+^ < 135 mmol/L	OR 2.70, *p* = 0.002	OR 3.03, *p* = 0.019	OR 1.63, *p* = 0.306	OR 1.29, *p* = 0.691
K^+^/0.5 mmol/L increase	OR 1.21, *p* = 0.051	OR 1.08, *p* = 0.573	OR 0.93, *p* = 0.660	OR 0.93, *p* = 0.662
K^+^ > 5.0 mmol/L	OR 2.78, *p* = 0003	OR 2.21, *p* = 0.127	OR 0.81, *p* = 0.743	OR 0.62, *p* = 0.562
Cre/0.5 mg/dL increase	OR 1.15, *p* = 0.003	OR 1.10, *p* = 0.170	OR 1.10, *p* = 0.089	OR 0.98, *p* = 0.844
Cre > 1.2 mg/dL	OR 7.08, *p* < 0.001	OR 6.86, *p* = 0.004	OR 1.54, *p* = 0.311	OR 0.69, *p* = 0.526
Urea/20 mg/dL increase	OR 1.26, *p* < 0.001	OR 1.27, *p* = 0.001	OR 1.09, *p* = 0.212	OR 0.94, *p* = 0.566
Urea > 100 mg/dL	OR 4.07, *p* < 0.001	OR 4.84, *p* = 0.001	OR 1.81, *p* = 0.245	OR 0.85, *p* = 0.804
Hs Troponin I > 20 (ng/L)	OR 8.88, *p* = 0.033	OR 8.88, *p* = 0.033	OR 1.88, *p* = 0.411	OR 0.98, *p* = 0.978
Hematocrit/5% increase	OR 0.89, *p* = 0.251	OR 0.99, *p* = 0.953	OR 0.79, *p* = 0.09	OR 0.83, *p* = 0.352
Hematocrit < 40%	OR 1.19, *p* = 0.567	OR 0.60, *p* = 0.286	OR 2.57, *p* = 0.043	OR 1.88, *p* = 0.375
WBC/2 × 10^3^/μL increase	OR 1.04, *p* = 0.498	OR 0.95, *p* = 0.577	OR 1.04, *p* = 0.565	OR 1.07, *p* = 0.515
WBC > 10^4^/μL	OR 1.04, *p* = 0.892	OR 0.54, *p* = 0.185	OR 2.21, *p* = 0.079	OR 2.17, *p* = 0.210
In-hospital Arrest	OR 13.53, *p* < 0.001	OR 9.00, *p* = 0.01	OR 2.17, *p* = 0.344	-
Killip category 4 vs. other	OR 12.07, *p* < 0.001	OR 9.13, *p* < 0.001	OR 2.23, *p* = 0.241	OR 1.77, *p* = 0.536
In-hospital infection	OR 7.54, *p* < 0.001	OR 6.80, *p* < 0.001	OR 6.27, *p* < 0.001	OR 6.42, *p* = 0.006
Blood cultures (+)	OR 7.50, *p* < 0.001	OR 6.76, *p* = 0.001	OR 3.41, *p* = 0.05	OR 3.92, *p* = 0.120
MCS	OR 8.17, *p* < 0.001	OR 8.00, *p* = 0.078	OR 2.13, *p* = 0.508	-
CVVHDF	OR 11.50, *p* < 0.001	OR 7.87, *p* < 0.001	OR 3.92, *p* = 0.06	OR 1.77, *p* = 0.536
IMV	OR 59.92, *p* < 0.001	OR 23.67, *p* < 0.001	OR 4.76, *p* = 0.017	OR 2.88, *p* = 0.203
Days in CICU > 2	OR 4.27, *p* < 0.001	OR 5.25, *p* = 0.002	OR 3.53, *p* = 0.004	OR 1.43, *p* = 0.549

ADHF = acute decompensated heart failure, BP = blood pressure, bpm = beats per minute, CAD = coronary artery disease, CICU = cardiac intensive care unit, COPD = chronic obstructive pulmonary disease, CVVHDF = continuous venovenous hemodiafiltration, GFR = glomerular filtration rate, IMV = invasive mechanical ventilation, MCS = mechanical circulatory support, OR = odds ratio, WBC = white blood cells.

**Table 3:** Multivariate associations of studied parameters with in-hospital and 30-day mortality in all patients and the ADHF subgroup.

**In-Hospital Analysis**		
	**All patients (*n* = 294)**	
	**OR (95% CI)**	***p* value**
In-hospital intubation	43.52 (14.57, 130.02)	<0.001
Systolic BP < 100 mmHg	4.78 (1.78, 12.83)	0.002
In-hospital infection	4.42 (1.60, 12.18)	<0.001
Coronary Artery Disease	4.20 (1.46, 12.10)	0.008
SatO_2_ < 90%	4.67 (1.26, 17.28)	0.021
Urea > 100 mg/dL	2.97 (1.06, 8.30)	0.038
	**ADHF subgroup (*n* = 92)**	
	**OR (95% CI)**	***p* value**
In-hospital intubation	68.39 (8.29, 564.08)	<0.001
In-hospital infection	9.29 (1.55, 55.68)	0.015
Urea > 100 mg/dL	10.55 (1.58, 70.42)	0.015
Coronary artery disease	10.01 (1.55, 64.90)	0.016
Mean BP < 60 mmHg	5.81 (1.16, 29.14)	0.033
SatO_2_ < 90%	11.73 (1.10, 125.46)	0.042
**30-Day Mortality for Discharged Patients**		
	**All patients (*n* = 233)**	
	**OR (95% CI)**	***p* value**
In-hospital infection	3.85 (1.43, 10.38)	0.008
ADHF subgroup	3.12 (1.24, 7.82)	0.015
PO_2_/FiO_2_ < 300	3.55 (1.21, 10.45)	0.021
	**ADHF subgroup (*n* = 65)**	
	**OR (95% CI)**	***p* value**
In-hospital infection	7.04 (1.68, 29.55)	0.008
Mean BP < 60 mmHg	6.67 (1.24, 30.46)	0.014

ADHF = acutely decompensated heart failure, BP = blood pressure.

## Data Availability

The data presented in this study are available on request from the corresponding author. The data are not publicly available due to privacy issues.
